# Comparing the production of complex sentences in Persian patients with post-stroke aphasia and non-damaged people with normal speaking

**Published:** 2016-01-05

**Authors:** Azar Mehri, Askar Ghorbani, Ali Darzi, Shohreh Jalaie, Hassan Ashayeri

**Affiliations:** 1 Department of Speech Therapy, School of Rehabilitation, Tehran University of Medical Sciences, Tehran, Iran; 2 Department of Neurology, School of Medicine, Tehran University of Medical Sciences, Tehran, Iran; 3 Department of Linguistics, School of Letters and Humanities, University of Tehran, Tehran, Iran; 4 Department of Physiotherapy, School of Rehabilitation, Tehran University of Medical Sciences, Tehran, Iran; 5 Department of Rehabilitation, School of Rehabilitation, Iran University of Medical Sciences, Tehran, Iran

**Keywords:** Stroke, Nonfluent Aphasia, Agrammatism, Production, Sentence

## Abstract

**Background:** Cerebrovascular disease leading to stroke is the most common cause of aphasia. Speakers with agrammatic non-fluent aphasia have difficulties in production of movement-derived sentences such as passive sentences, topicalized constituents, and Wh-questions. To assess the production of complex sentences, some passive, topicalized and focused sentences were designed for patients with non-fluent Persian aphasic. Afterwards, patients’ performance in sentence production was tested and compared with healthy non-damaged subjects.

**Methods:** In this cross sectional study, a task was designed to assess the different types of sentences (active, passive, topicalized and focused) adapted to Persian structures. Seven Persian patients with post-stroke non-fluent agrammatic aphasia (5 men and 2 women) and seven healthy non-damaged subjects participated in this study. The computed tomography (CT) scan or magnetic resonance imaging (MRI) showed that all the patients had a single left hemisphere lesion involved middle cerebral artery (MCA), Broca`s area and in its white matter. In addition, based on Bedside version of Persian Western Aphasia Battery (P-WAB-1), all of them were diagnosed with moderate Broca aphasia. Then, the production task of Persian complex sentences was administered.

**Results:** There was a significant difference between four types of sentences in patients with aphasia [Degree of freedom (df) = 3, P < 0.001]. All the patients showed worse performance than the healthy participants in all the four types of sentence production (P < 0.050).

**Conclusion:** In general, it is concluded that topicalized and focused sentences as non-canonical complex sentences in Persian are very difficult to produce for patients with agrammatic non-fluent aphasia. It seems that sentences with A-movement are simpler for the patients than sentences involving A`-movement; since they include shorter movements in compare to topicalized and focused sentences.

## Introduction

Cerebrovascular disease leading to stroke is the most common cause of aphasia.^1^ Broca’s aphasia is one of the non-fluent aphasia types that were characterized by the symptoms such as slow speech rate, effortful production, short phrases, restricted speech output, short sentences, and simple structured sentences and agrammatism. The agrammatic speech patterns include the use of the subject-verb-object (in English) structure.^2^

Agrammatic speakers have difficulties in the production of movement derived sentences such as passive sentences, object relative clauses, object clefts, topicalized constituents, Wh-questions, and even yes/no questions (in languages that require movement to higher nodes in these constructions),^3^^-^^13^ but they have better performance in the production of canonical structures.

The basic word order in Persian is subject-object-verb and it is called the canonical word order, any changes in this constituent ordering or any kind of movement of constituents from their base position results in what is called non-canonical structure.

In the linguistic tradition, the structure of sentences has long been represented in the form of tree diagrams. Several hypotheses have been proposed with regard to syntactic movement in agrammatism. Friedmann and Grodzinsky^14^ supported Hagiwara’s hypothesis^15^ of hierarchical degradation of the syntactic tree structure and argued that complementizer phrases (CPs) always impair in agrammatism because they are the highest projections. They named their hypothesis “tree pruning hypothesis.” Bastiaanse and van Zonneveld^16^ proposed derived order problem hypothesis. According to their hypothesis, overt movement of any constituent (including verbs) in a sentence resulting in a derived order is difficult for Dutch agrammatic speakers, regardless of the landing site of the moved constituent in the syntactic tree.

Thompson et al.,^17^ stated the complexity account of the treatment efficacy based on experimental studies. In several intervention studies, Thompson et al.^12^^,^^13^^,^^17^^,^^18^ found that complex sentences is difficult for agrammatic patients. They have shown that training more complex forms facilitates learning of less complex structures. These hypotheses were tested on patients with agrammatic aphasia speaking different languages, such as English,^19^ Japanese,^20^ Korean and Spanish,^21^ German,^22^^,^^23^ Indonesian,^24^ Dutch,^16^ Turkish,^25^^-^^27^ Russian,^28^ Italian,^29^ and Greek.^30^ In general, it was found that production of complex structures is difficult for agrammatic patients.

In this present study, we used the basic assumptions of Minimalist Program as elaborated in Chomsky.^31^ According to this syntactic model, there are two kinds of phrasal movements that are derived from the general rule of “move-alpha”: Noun phrase-movement (NP-movement) and Wh-question movement (Wh-movement). NP-movement is involved in passives and raising constructions and Wh-movement is involved in Wh-questions or relativization. Production of sentences that involve these movements are difficult for agrammatic patients. Hence, it is necessary that sentences were designed which have been involved two movements. A`-movement is a position that elements move into spec of CP and this position do not participate in theta role assignment, but A-movement is land that elements move into spec of TP and this position participate in theta role assignment.

The aim of the study is assessing complex sentences production in Persian aphasic patients. Based on this aim, topicalized and focused sentences of A`-movement and passive sentences of A-movement were designed in Persian non-fluent aphasic patients. We supposed these sentences were non-canonical and complex sentences in Persian. In this study, a task should be designed to assess different types of sentences that were adapted to Persian structures. Production of four type sentences (active, passive, topicalized and focused sentences) was assessed in Persian agrammatic patients using this task. Then, patients’ performances were compared with normal subjects.

## Materials and Methods

In this cross-sectional study, 14 individuals including seven Persian stroke individuals with non-fluent agrammatic aphasia (5 males, 2 females; mean age = 54.28 years) and seven healthy normal subjects (5 males, 2 females; mean age = 53.85 years) participated in this study. computed tomography (CT) scan or magnetic resonance imaging (MRI) showed all patients had a single left hemisphere lesion that involving middle cerebral artery (MCA), Broca’s area and in its white matter. They were right-handed and had left hemiplegia. Cerebral dominancy in all participants was left hemisphere. Post-stroke time was between 2 and 13 years. None of the subjects had a history of prior neurological or psychiatric disorders, developmental speech and language disorders and drug or alcohol abuse. All of the individuals were native Persian speakers and had normal or corrected-to-normal hearing visual acuity. All patients had mild or no apraxia (based on Yadegari^32^). If each patient would not like to enter this study, he/she was omitted. Aphasic subjects were selected from rehabilitation and speech therapy centers of Tehran University of Medical Sciences and Iran University of Medical Sciences, Iran. Seven aged-matched individuals (5 males, 2 females; mean age = 53.85 years) with no neurologic impairment served as control group. All participants had, at least, a high-school education (mean = 12 years). Aphasic demographic data are given in table 1.

***Language testing***

The aphasia type was determined non-fluent based on the Bedside version of Persian Western Aphasia Battery (P-WAB-1)‎,^33^ scores of speech fluency were between 3 and 5 and aphasia question (AQ) was between 59.2 and 77.5. According to Nilipour et al.^34^ study our patients were placed at moderate AQ range. 

Noun naming was measured by the picture naming test^35^ (range: 55.04-94.50%, mean = 74.31%) and patients’ performance was better than verb naming which was assessed by the picture verb naming test^36^ (range: 50.00-82.57%, mean = 63.95%). A speech therapist assessed all of language tests for aphasia subjects.

***Experimental stimuli***

We required Persian verbs and sentences for designing production task. Based on their occurrence of written frequency (range = 1-39522 per approximately 10 million),^37^ 32 semantically reversible, two arguments, imageable verbs were selected which included 8 simple and 24 complex transitive verbs. Finally, 20 verbs were selected from a questionnaire and then 15 individuals including speech therapists and linguists were asked to score and confirm appropriate verbs for aphasic subjects. In this way, content validity for verbs obtained 63.87-94. Two animate nouns were selected for sentences based on their occurrence of written frequency (range = 821-5729 per approximately 10 million).^37^ Content validity for nouns obtained 82.33-94. For each verb, four sentence types were designed and for each sentence; two black and white pictures were drawn by a graphist. One picture was depicted the target sentence and the other one, the foil sentence. Face validity for pictures obtained 85-96.67 by scoring speech therapists and linguists. The roles of subjects and objects were reversed in the target and the foil sentences. Four sentence types were composed of active, passive (A-movement), topicalized and focused sentences (A`-movement) for each verb (totally, 80 sentences).

***Sentence production***

The task contained 20 sentences for each four types of sentences (active, passive, topicalized and focusing). Production of the 80 sentences was assessed using a sentence production priming paradigm.^12^ In this way, the examiner modeled the production of one type of sentence using the foil picture. Then participants were asked to produce a sentence with the same structure using the target picture. For example, the examiner produced a passive sentence with the foil picture, and the participant produced a passive sentence with the target picture which had the reverse subject and object. The 80 stimulus were presented to participants randomly, and their responses were written and recorded with audiotape, simultaneously. Scoring was based on correct response and responding time was 10 seconds. No feedback was presented for a correct or non-correct response. Each correct or incorrect response was checked and confirmed by a linguist, one more time. For example, the omission of morphological elements and word substitutions were considered correct. 

Notably, this study was permitted by Ethics Committee of Tehran University of Medical Sciences with 93/130/1639 codes and the patient or his/her partner signed the study inform consent.

In this study all data were quantitative, therefore, to report findings of production of four types sentences, descriptive statistics was used to calculate percentages and means (standard deviation) of scores. Non-parametric statistics was utilized to analyze data since the data does not have a normal distribution.

The non-parametric Mann–Whitney U was used to compare the production of each four types of sentences between patient and control groups and within each group Friedman test and post-hoc Wilcoxon test by adjusted P value was used. In addition, we analyzed the production performance of two groups by these tests. The significance level was P < 0.050.

**Table 1:** Aphasic participants data (n = 7)

**Name**	**Age (year)**	**Gender**	**Education (year)**	**Post onset time (year)**	**Lession**	**Etiology**
BH	62	M	12	3	Left insula-putamen	CVA
SP	50	M	12	2	Left fronto-temporal	CVA
MP	65	M	12	1	Left fronto-temporal	CVA
SN	59	M	14	5	Left putamen	CVA
MY	53	M	12	13	Left fronto-tempo-pariatal	CVA
AR	43	F	16	9	Left fronto-temporal	CVA
MM	48	F	9	5	Left fronto-tempo-pariatal	CVA

M: Male; F: Female; CVA: Cerebrovascular accident

**Table 2:** Aphasic subjects’ language test data

**Number of subjects**	**S1**	**S2**	**S3**	**S4**	**S5**	**S6**	**S7**
P-WAB-1							
Spontaneous speech content (n = 10)	6	6	5	6	5	7	6
Fluency of spontaneous speech (n = 10)	5	4	3	5	4	5	5
Auditory comprehension (n = 10)	10	6	10	10	10	9	10
Sequential commands (n = 10)	7	6	7	9	9	9	10
Naming (n = 10)	8	6.5	9	7	6	8.5	7
Repetition (n = 10)	10	7	7	7	9	8	8
AQ = 100	76.6	59.2	68.3	73.3	71.6	77.5	76.6
Syntactic comprehension of BAT (%)	90.80	58.62	74.71	91.95	81.61	67.81	80.46
Object naming (%)	69.72	55.04	61.46	78.90	86.23	94.50	74.31
Verb naming (%)	68.18	50	60.60	82.57	61.36	61.36	63.63
Sentence production (%)							
Active sentence	60	85	70	80	60	80	85
Passive sentence	70	55	35	65	20	5	25
Topicalized sentence	20	35	0	30	0	0	15
Focused sentence	15	0	0	40	0	0	15

P-WAB-1: Persian Western Aphasia Battery; AQ: Aphasia quotient; BAT: Bilingual aphasia test

## Results

***Sentence production***

The results showed all aphasic patients had more difficulties in the production of passive, topicalaized and focused sentences (Table 2). Their score ranges were 5-70% (mean = 39.28%), 0-35% (mean = 14.28%), 0-40% (mean = 10%) for passive, topicalaized, and focused sentences, respectively. However, the best performance was obtained for active sentence production (mean = 74.28%). Table 3 indicates that there is a significant difference between four types of sentence in aphasic individuals [Degree of freedom (df) = 3, P < 0.001]. No error was produced by the control group, and their data were not presented. 

All aphasic participants showed worse performance than the normal participants in the total of four types of sentence’s production. Significant differences were shown in table 4.

**Table 3:** Statistical analysis comparing the production of all sentence types together in patients

**Production**	**df**	**P** *
Active sentence	3	< 0.001
Passive sentence		
Topicalized sentence		
Focused sentence		

df: Degree of freedom

* Kroskal–Wallis test, P value level < 0.050

## Discussion

In this study, we assessed the production of canonical and non-canonical (complex) sentences in Persian post-stroke non-fluent aphasic patients. We compared patients’ performance to healthy subjects without brain damage by a task that designed to assess four types of sentence’s structures. 

**Table 4:** Comparison of sentence production between patient and normal participants

**Types of sentence** **‎**	**Z**	**P** *
Active sentence production		
Normal		
Patients	-3.356	0.001
Passive sentence production		
Normal		
Patients	-3.343	0.001
Topicalized sentence production		
Normal		
Patients	-3.360	0.001
Focused sentence production		
Normal		
Patients	-3.390	0.001

* Mann–Whitney U-test, P value level < 0.050

Results showed that the production of active, passive, topicalized, and focused sentences is difficult for aphasic patients. However, the patients perform better in active sentences than the other types. In our task, two types of sentences were designed based on A`- movement structure (i.e., topicalized and focused sentences) and one sentence was designed based on A`-movement structure (i.e., passive sentences). The findings indicated that producing focused sentences were worse than topicalized ones in patients while passive sentences were better than topicalized and focused sentences. Nevertheless, there was a significant difference between productions of all four types of sentences.

In general, it is concluded that topicalized and focused sentences are non-canonical and complex sentences in Persian language, and their production is significantly difficult for agrammatic non-fluent aphasic patients. Several authors studied similar sentence structures in different languages and obtained results correspond to our findings for agrammatic subjects.^6^^,^^7^^,^^12^^-^^14^^,^^23^^-^^28^^,^^30^^,^^38^

When the production of each four types of sentences between patients, and healthy groups were compared, significant differences were found. That is, agrammatic patients have more difficulties in the production of A-movement and A`-movement structures than control subjects. The study of Thompson and Shapiro,^12^ reveals that agrammatic individuals rarely produce complex sentences, containing Wh-movement or embedded clausal structures. Persian aphasic patients had more difficulties in the production of complex sentences such as passive, topicalized and focused sentences. Furthermore, in production of passive sentences Persian agrammatic speakers performed better than topicalaized and focused sentences. It seems that A-movement structures are simpler than A`-movement structures in Persian because the length of passive sentences is shorter than topicalaized and focused ones. Active sentences are a canonical sentence and basic word order in Persian language, so both groups performed well in active sentences.

As be mentioned obviously, several hypotheses have been proposed with regard to syntactic movement in agrammatism. All of them state that production of sentences with longer movement in syntax tree is a more difficult than shorter movement and without movement in this tree. Therefore, findings in Persian agrammatic patients are accountable to these hypotheses in syntactic movement. 

It should be noted that the control subjects without brain damage responded correctly to produce all kinds of sentences. These findings suggest that our task is suitable to elicit the production of all types of sentences for adults and it can elicit the sentences level production problems in agrammatic aphasic patients.

Although there are a lot of studies in the field of aphasic patients in other languages, there is lacking information about language abilities of this group in Persian to guide speech and language therapists during assessment and treatment planning. This finding helps to clinicians that improve production of sentences in non-fluent aphasia patients. Interventional findings show that training complex sentences not only improves production of these sentences but also simultaneously improves simpler structures. Furthermore, fewer treatment sessions are required for participants who receive treatment on complex forms first.

We suggest more research in finding of types of A-movement and A`-movement sentences in Persian. Furthermore, clinicians train types of these sentences to patients in the experimental studies.

## Conclusion

It seems that sentences with A-movement are simpler for the patients than sentences involving A`-movement as they include shorter movements than topicalized and focused sentences. To conclude, our findings in Persian agrammatic patients correspond to Chomsky’s theory (Minimalist Program).

## Notes:

**How to cite this article:** Mehri A, Ghorbani A, Darzi A, Jalaie Sh, Ashayeri H. Comparing the production of complex sentences in Persian patients with post-stroke aphasia and non-damaged people with normal speaking. Iran J Neurol 2016; 15(1): 28-33**.**
